# Supplementary material for: COPI selectively drives maturation of the early Golgi
Source: eLife. 2015 Dec 28;4:e13232. doi: 10.7554/eLife.13232 (PMC4758959; doi:10.7554/eLife.13232)
Supplement: Supplementary file 2. — DOI: http://dx.doi.org/10.7554/eLife.13232.039 [file elife-13232-supp2.zip › 4D Imaging Protocols and Plugins/Editing 4D Movies.docx]

**Editing 4D Movies with ImageJ Plugins**

The goal is to edit a 4D movie to display only one or a few spots. The edited movies can then be analyzed to quantify fluorescence time courses and marker colocalization.

As the first step in the procedure, examine a movie from a deconvolved 4D series to identify candidate structures that can potentially be tracked over much or all of their lifetimes. Then perform the following steps using our custom “4D Movies” plugins.

Make Montage Series

1. Run this plugin, and open a 4D 8-bit TIFF hyperstack from a confocal. This data set should already be deconvolved, bleach corrected, and cropped.

2. Choose a scale factor for magnifying the individual slice images. The default value is 4.0, but you can choose another number as long as the montage will fit on the screen.

3. Choose the slices that will be used to create the montage. You can omit slices that have no fluorescence data. (Alternatively, such slices can be removed ahead of time by editing the hyperstack in ImageJ.)

4. Allow the montage series to be created. The plugin cannot be aborted during scaling, but can be aborted using Esc during assembly of the montage series.

5. Save the montage series as a file, which will serve as the “original” montage series during the editing phase.

Edit Montage Series

1. Run this plugin, and open the original montage series file.

2. The first time you work with a given montage series, choose the option “Create New Montage”, which will duplicate the original montage series.

If you have already begun editing the montage series, use “Open Existing Montage”, and choose the edited montage series.

In either case, the edited version of the montage series will be displayed on top of the original version.

3. Use the keyboard commands listed below to work with the original and edited montage series. You can also use the sliders at the bottom of the window. It is recommended to use these controls whenever possible rather than the standard ImageJ menu commands and image adjustment dialogs.

a) Use Tab to switch between the original and edited montage series windows. These two windows should stay synchronized with respect to channel, time point, and display parameters.

b) Use the Left Arrow and Right Arrow keys to move between time points. You can also drag the lower slider.

c) Use Cmd or Opt together with the Left Arrow and Right Arrow keys to switch between channels. You can also drag the upper slider.

d) Use the Up Arrow and Down Arrow keys to increase or decrease the display brightness of the currently selected channel.

Use Cmd or Opt together with the Up Arrow and Down Arrow keys to jump to either the default brightness, or the maximum or minimum brightness, depending on the current brightness setting of the currently selected channel.

e) Use the R, G, and B keys (uppercase or lowercase) to toggle visibility of the red, green, and blue channels, respectively.

f) Use the Z key to replace the edited contents of the currently displayed channel and time point in the edited montage series with the original contents.

Note that Z is not a generic Undo key. This replacement function is irreversible.

g) Use the A key to select the entire image.

h) Use the E key to erase all of the fluorescence in the selected channel going forward or back in time. You will be prompted to choose the direction.

i) To delete everything except the desired spot, first select the spot in each of the relevant slices by drawing regions of interest (ROIs). A fast method for drawing ROIs is described below in (j).

You can use Shift to add pixels to an existing ROI, or Opt to trim pixels from an existing ROI.

When you are satisfied with the ROI, press the Delete key. For the currently selected channel, the nonselected pixels outside the ROI will be deleted.

If you make a mistake, reverse the procedure by pressing Z.

If you want to delete the selected pixels inside the ROI, use Cmd or Opt together with the Delete key. If nothing is selected, the entire image will be deleted.

j) To automatically trace a spot in all of the relevant slices, do the following. First, select the spot by drawing a crude ROI. Make sure the appropriate channel is selected. Then press Return. The spot will be outlined in the original slice and in the adjacent slices.

If you change the brightness using the Up Arrow and Down Arrow keys, and then repeat the automatic tracing, the borders will expand or contract accordingly.

If the automatic tracing captures too much or too little, you can add to the selection using the Shift key or remove part of the selection using the Opt key.

4. Periodically save the edited montage series. The S key will work for this purpose. You can return to the edited file later if you wish to make additional edits.

Montage Series to Hyperstack

1. Open an edited montage series.

2. Run this plugin. Choose the desired time points, and generate a hyperstack.

3. This hyperstack can be projected to make a 4D movie. In addition, it can be quantified using the Analyzed Edited Movie plugin described below.

Analyze Edited Movie

1. Open a hyperstack generated from an edited montage series.

2. Run this plugin. You will need to enter the time interval between Z-stacks.

3. The results will be displayed in a window.

“Red” and “Green” are the total signals from the Z-stacks at each of the indicated time points.

“Red Masked” and “Green Masked” are the red signal that shines through the green mask and vice versa. These masks are created for each slice in a Z-stack, so they account for colocalization along all three axes.

4. If you click on this window and then save, the result will be a file that can be opened by Excel. You can then process the data in two ways:

a) To show a plot of the red and green signals over time, plot the Red and Green data as a function of Time.

b) To quantify colocalization, use Excel to sum the signals in each column except Time. Then the average red signal colocalizing with green is the (Red Masked/Red) ratio, and the average green signal colocalizing with red is the (Green Masked/Green) ratio.
