# Supplementary material for: COPI selectively drives maturation of the early Golgi
Source: eLife. 2015 Dec 28;4:e13232. doi: 10.7554/eLife.13232 (PMC4758959; doi:10.7554/eLife.13232)
Supplement: Supplementary file 2. — DOI: http://dx.doi.org/10.7554/eLife.13232.039 [file elife-13232-supp2.zip › 4D Imaging Protocols and Plugins/Processing 4D Movies for Presentation.docx]

**Processing 4D Movies for Presentation with ImageJ**

The goal is to convert an edited 4D movie to a form that is suitable for presentation or publication. Starting with an 8-bit 4D hyperstack created from a montage series, perform the following operations.

Convert to 16-Bit

This conversion prevents data loss during the subsequent average projection.

1. Choose Image > Color > Split Channels.

2. For each of the resulting three windows, choose Image > Type > 16-bit. Wait for the conversion to finish.

3. Choose Image > Color > Merge Channels.

4. Choose Process > Math > Multiply. For the multiplication factor, use 256.

5. OPTIONAL: Choose Image > Adjust > Brightness/Contrast. For each of the three channels in turn, press the Auto button in the B&C window. You can adjust the channels by dragging the “c” slider in the 4D hyperstack window. The images should look normal once again, but you can adjust the Minimum and Maximum values if desired.

6. If desired, save the 16-bit 4D hyperstack with a new name. Wait for the saving to finish. Note that the TIFF file will have doubled in size.

Merge if Desired

1. If you wish to merge the fluorescence from two 4D movies, use the custom Merge Two Hyperstacks plugin with the first option selected.

2. If you wish to place one 4D movie above the other—e.g., an original movie above an edited one—use the custom Merge Two Hyperstacks plugin with the second option selected.

Average Project

1. Choose Image > Stacks > Z Project. If desired, you can select a subset of each Z‑stack by choosing the Start and Stop slices. Choose “Average Intensity”, and press OK. Wait for the projection to finish.

2. If desired, adjust the brightness and contrast of the individual channels as described above. You can use the “Set” button to specify the minimum and maximum values.

3. To add a time stamp, use Image > Stacks > Label. Use the format 00:00, with the appropriate time interval in seconds. To place the label in the lower left corner, set the Y location to be 1 less than the Y value of the lowest row of pixels in the image.

4. Save this file. Wait for the saving to finish.

Make a Movie

1. Choose File > Save As > AVI. For the compression, choose PNG. A frame rate of 10 fps is typically suitable. Press OK.

2. Use QuickTime Player 7 to export the AVI file as a much smaller MOV file.
