# Supplementary material for: COPI selectively drives maturation of the early Golgi
Source: eLife. 2015 Dec 28;4:e13232. doi: 10.7554/eLife.13232 (PMC4758959; doi:10.7554/eLife.13232)
Supplement: Supplementary file 2. — DOI: http://dx.doi.org/10.7554/eLife.13232.039 [file elife-13232-supp2.zip › 4D Imaging Protocols and Plugins/Taking 4D Movies on the Leica SP5.docx]

**Taking Movies on the Leica SP5**

Preparing the Cells

- Inoculate a yeast culture from a healthy preculture, and grow overnight in 5 mL nonfluorescent minimal medium (NSD) in a 50-mL baffled flask. Aim to image the cells at an OD_600_ of ~0.5.
- Use a MatTek dish with a high precision 0.170 mm coverglass bottom. Prepare the dish with ConA: pipette 250 µL ConA onto the dish, wait 15 min, wash thoroughly with dH_2_O, and let dry.
- Just before imaging, adhere cells to the dish: pipette 250 µL from your culture onto the dish, wait 10 min, and rinse gently several times with NSD by pipetting. Leave 2-3 mL of fresh NSD in the dish.

Imaging Parameters

These parameters can be adjusted as necessary, but they are good starting points, especially for low signals:

- 100x or 63x objective (NA=1.4 or higher)
- IMPORTANT: the DIC prism should not be in the light path or the fluorescence images will be sheared and distorted
- IMPORTANT: with the 100x objective on the Leica STED, the quarter wave plate should be in place or the red and green signals will be offset in Z
- Frame size: 256 x 128 (width x height)
- Zoom: 9
- Pixel size: 60-70 nm (resulting from the given frame and zoom)
- Scan direction: bidirectional, phase = 3.15, but judge from the brightfield image whether horizontal lines are aligned
- Pinhole: 1.2 AU
- Line averaging: 8
- Slice thickness: approximately 0.3 µm
- Time interval per stack: 2 s
- Laser settings:
  - 488: 3-10%, HyD, collection window = 495-550 nm, gain = 400-500
  - 561: 3-10%, HyD, collection window = 575-750 nm, gain = 400-500
- PMT for brightfield, gain = 300-350

Notes:

- A taller frame takes longer to scan. A wider frame enables smaller pixels with almost no cost in speed, but smaller pixels may cause increased photobleaching and are usually unnecessary for movies. A pixel size of 60‑70 nm seems best.
- Line averaging of 8 is the highest possible while maintaining ≤ 2 s intervals, as long as your Z-stack is ~24 slices or fewer. Line averaging makes a *big* difference when the signal is low.
- Generally, one must find a compromise to get a decent signal with low bleaching. If the laser power is so low that you have difficulty seeing the signal in the preview, your movies may be unusable, but don’t be overly concerned about how nice the images look during collection. If possible, reduce the laser power to 5% or even lower. Bleaching increases nonlinearly with laser intensity, so by reducing the laser power, you may start with a weaker signal but obtain a much longer time window of usable data. Note also that the 488 laser can bleach mCherry as well as GFP, so minimizing the power of this laser will prolong the signal for both channels.

Filtering

For weak signals, deconvolution may cause flickering that makes the analysis impossible. In this case, it sometimes helps to pre-process the data with a hybrid median filter using our ImageJ plugin.

1. We find that one iteration with the 3D hybrid median often works well, but if it removes too much data, try one iteration with the standard 2D hybrid median.

2. After hybrid median filtering, do a 2D Gaussian blur with a 1-pixel radius using the built-in ImageJ command.

Deconvolution

1. Go to Applications > SVI_old > Huygens Essential X11. Open your raw .lif file, or a filtered .tif file if the images were pre-processed.

2. Go to Deconvolution > Parameter wizard. If you are using a raw .lif file, it will automatically supply the NA, excitation/emission, pixel size, step size, and wavelengths. Click the “Next” arrows to proceed through the wizard. Accept all of the values from the metadata, except:

- Change the embedding medium refractive index to 1.35.
- Change the backprojected pinhole radius to 342 nm for all channels.

If you are using a pre-processed .tif file, you will need to enter all of the parameters manually.

3. Now run the deconvolution wizard, clicking the “Next” arrows to proceed through the wizard. Do not load a template. Crop the image as desired. Compute the logarithmic mapping function, then click “Next”.

4. For the background, try “Auto” first. Click “Accept”. Leave the values for maximum iterations, etc., but change the signal-to-noise ratio to 10. Click “Deconvolve.”

6. If the deconvolved image is satisfactory (eliminates background while maintaining every signal you would consider a spot), click “Accept, to next channel.” If there is a lot of noise, click “Restart channel.” Click “Manual” to input a background value. Begin with low background values (2-3) and increase only if necessary.

7. Repeat for the other fluorescence channel. When you reach the brightfield channel, click “All done.” Don’t deconvolve the brightfield image.

8. For each channel, choose the appropriate deconvolved or original image series, and click “Next”. It is convenient to set red as Ch0, green as Ch1, and brightfield as Ch2. Skip the Z‑drift correction. Click “Done”.

9. Select the deconvolved image and go to File > Save As > Tif 8 bit. Save to “One” file per channel in a new “decon” folder.

ImageJ

1. In ImageJ, go to File > Import > Image Sequence. Select your “decon” folder. Accept the options.
2. Go to Image > Hyperstacks > Stack to Hyperstack (shortcut=Shift+L). Choose order “xyzct” and input the appropriate number of channels, slices per stack, and frames. Click “OK”.
3. To correct for bleaching (recommended), go to Image > Color > Split Channels (shortcut=8). Select the red channel, then go to Plugins > EMBLtools > Bleach Correction (shortcut=6). Choose “Eponential Fit (Frame-wise)” as your correction method. Click “OK”. Make sure the graph shows a good fit, then close the graph window and log window and original channel window, keeping the new DUP_... window. Repeat to correct the other fluorescence channel, but leave the brightfield channel as is.
4. Go to Image > Color > Merge Channels (shortcut=U). Place the channels in their proper slots and click “OK”. The movie is now ready to adjust/project/montage.
